# A New Ground-Based Pseudolite System Deployment Algorithm Based on MOPSO

**DOI:** 10.3390/s21165364

**Published:** 2021-08-09

**Authors:** Wenjie Tang, Junping Chen, Chao Yu, Junsheng Ding, Ruyuan Wang

**Affiliations:** 1 Shanghai Astronomical Observatory, Chinese Academy of Sciences, Shanghai 200030, China; tangwj@shao.ac.cn (W.T.); yuchao@shao.ac.cn (C.Y.); dingjunsheng@shao.ac.cn (J.D.); wangry@shao.ac.cn (R.W.); 2 School of Astronomy and Space Science, University of Chinese Academy of Sciences, Beijing 100049, China

**Keywords:** pseudolite, multi-objective optimization, particle swarm optimization, visibility, dilution of precision

## Abstract

Pseudolite deployment is the premise of ground-based pseudolite system networking, which affects the coverage and positioning accuracy of ground-based pseudolite systems. Optimal deployment algorithms can help to achieve a higher signal coverage and lower mean horizontal precision factor (HDOP) with a limited number of pseudolites. In this paper, we proposed a multi-objective particle swarm optimization (MOPSO) algorithm for the deployment of a ground-based pseudolite system. The new algorithm combines Digital Elevation Model (DEM) data and uses the mean HDOP of the DEM grid to measure the geometry of the pseudolite system. The signal coverage of the pseudolite system was calculated based on the visual area analysis with respect to reference planes, which effectively avoids the repeated calculation of the intersection and improves the calculation efficiency. A selected area covering 10 km×10 km in the Jiuzhaigou area of China was used to verify the new algorithm. The results showed that both the coverage and HDOP achieved were optimal using the new algorithm, where the coverage area can be up to approximately 50% and 30% more than using the existing particle swarm optimization (PSO) and convex polyhedron volume optimization (CPVO) algorithms, respectively.

## 1. Introduction

GNSS has the advantages of globalization and all-weather operation, but in nonopen environments such as tunnels, indoors, and urban canyons, it is difficult for users to receive signals from more than four satellites at the same time due to the weak satellite signals, resulting in a decreased user positioning accuracy and even positioning failure [1,2,3,4]. The ground-based pseudolite system is an augmented system for GNSS positioning. Ground-based pseudolite systems can increase the number of visible satellites, improve the geometry of satellites, and consequently improve the user positioning accuracy [5,6,7,8,9]. Moreover, the ground-based pseudolite system can work independently in the special cases when a GNSS signal is unavailable [10].

Pseudolite deployment is the premise of ground-based pseudolite system networking, which affects the coverage and positioning accuracy. In recent years, the research on pseudolite system deployment has mainly focused on the geometry of the base station in special scenarios, and simulation experiments are also ideal situations where occlusion is not considered. Meng et al. (2007) analyzed the positioning accuracy under different constellations and different numbers of pseudolites, but there is a lack of discussion on the design of pseudolites constellations [11]. Sang et al. (2013) analyzed that the volume of the polyhedron formed by pseudolite base stations and receivers is approximately inversely proportional to the DOP, and they proposed the geometric configuration that should be avoided in the design of an independent pseudolite system, but did not give the ideal scheme of pseudolite station distributions [12]. Song et al. (2013) combined the tetrahedral volume method to improve the geometry of the base stations by constructing the best observation matrix, and they gave the best distribution of four pseudolites but did not consider the increase in the number of pseudolites [13]. Fan et al. (2014) proposed an experience-based search method, which has a certain optimization effect, but with randomness [14]. Kelly et al. (1990) proposed a ridge regression algorithm to reduce the global mean square error of positioning results, but this is not the optimal solution for users [15]. Shao et al. (2017) proposed a distribution method based on the particle swarm optimization (PSO) algorithm considering user location information, which can improve the positioning accuracy, but the proposed distribution strategy does not consider the actual environment [16].

The goal of stations deployment is to obtain the highest signal coverage and the lowest mean horizontal precision factor (HDOP) with a limited number of pseudolites. To solve these problems, this paper proposed an MOPSO algorithm for the deployment of a ground-based pseudolite system. Combining with DEM data of the deployed region, this algorithm uses the mean HDOP of the DEM grid to measure the geometry of the pseudolite system. The service range and positioning accuracy of the pseudolite system were optimized by the MOPSO algorithm at the same time. Section 2 briefly introduces the key factors to measure the distribution of the pseudolite system. Section 3 constructs the mathematical model of the pseudolite distribution problem and proposes an MOPSO algorithm to optimize the problem. Section 4 compares the MOPSO algorithm with other algorithms through a specific simulation experiment. Section 5 summarizes the main conclusions.

## 2. Overview of Pseudolite System

**Figure 1:**
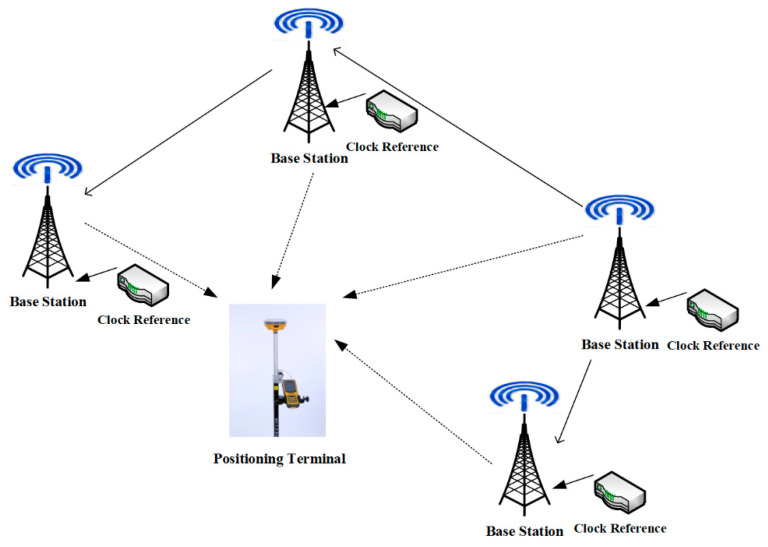
Schematic diagram of pseudolite system.

The regional ground-based pseudolite system is mainly composed of four parts: networking pseudolites (base station), pseudolite monitoring station, ground-based navigation signal network operation management system, and corresponding user terminals (as shown in Figure 1). The system can be understood as fixing the navigation satellites to the ground. The coordinates of the base station (pseudolite) are precisely measured in advance and are broadcast in the navigation message. The principle of the pseudolite system is similar to that of a GNSS system. The base station transmits navigation signals, which are received by user receivers to calculate the distance between the user receiver and the base station. The position of the user receiver can be obtained when signals of four or more pseudolites are received. A ground-based pseudolite system needs at least four base stations to provide four-dimensional spatiotemporal services [17,18].

### 2.1. Visual Area Analysis

According to the positioning principle of ground-based pseudolites, the pseudolite user receiver needs to receive signals from at least four pseudolites to realize the user’s positioning service. Although the signal power of pseudolites is much stronger than GNSS satellites, it may not be able to penetrate obstacles such as buildings. Therefore, to achieve the best service of a pseudolite system, we need to ensure larger areas where four or more ground-based pseudolites are simultaneously visible.

**Figure 2:**
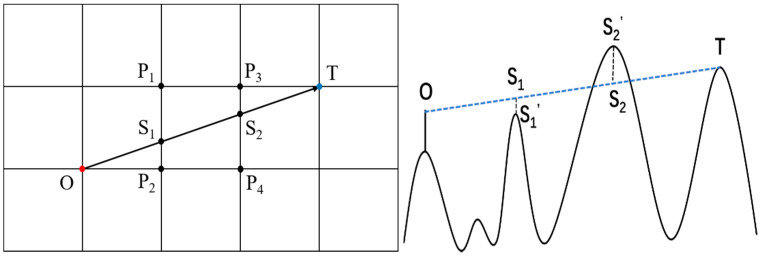
A common algorithm for visual domain analysis based on line of sight (**left**). Schematic diagram of occlusion in elevation direction (**right**).

The visual area depends on both the location of pseudolites and local geospatial information. To obtain the highest signal coverage of a region, algorithms for the deployment of pseudolites have been proposed. Among them, DEM-based visual range analysis is most used [19]. The basic methods are described below. Figure 2 shows the intersection points between the line of sight formed by the viewpoint (O) and the target point (T) and the DEM grids: points S_1_ and S_2_. The elevations of S_1_^′^ and S_2_^′^ are interpolated by the known elevations of DEM grids (P_1_, P_2_, P_3_, and P_4_). If the elevation of S_1_^′^ or S_2_^′^ is higher than the height of line of sight (LOS), the propagation is blocked, and the target point is invisible.

**Figure 3:**
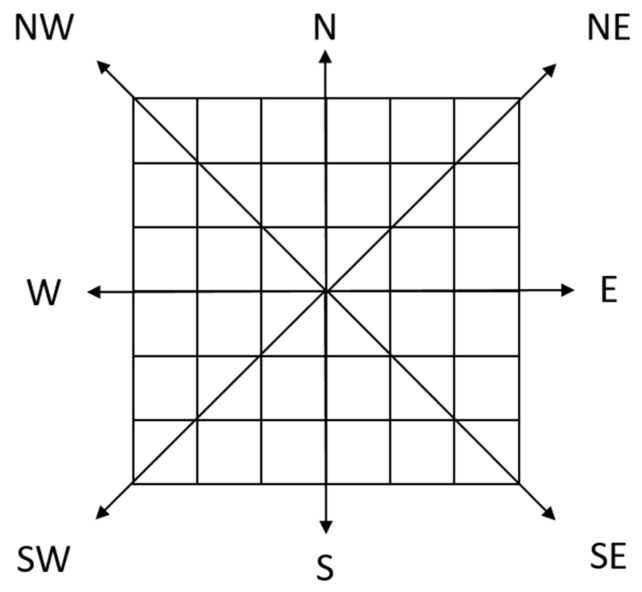
DEM regional division diagram.

Although the above method is simple in logic, there are a lot of repeated calculations and data redundancy. The processing time greatly increases, and the processing efficiency greatly decreases when the DEM grid data sampling increases. To solve the problem, we changed the strategy from judging the individual LOSs to the visual situation between the corresponding plane, formed by the target point and the viewpoint, and all the points in the target area. This algorithm does not need DEM interpolation and does not have any repeated calculation, so it can greatly improve the computation efficiency. The steps of the procedure are introduced as follows:

Step 1: As shown in Figure 3, we define the viewpoint as the coordinate origin *O*, and all of the DEM grids are divided into 8 linear directions and 8 corresponding sector areas.

**Figure 4:**
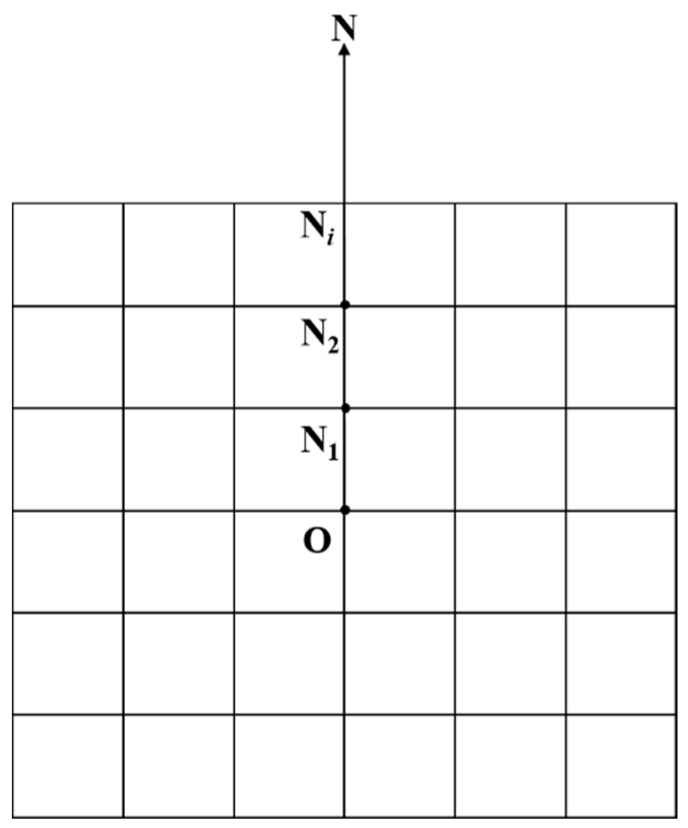
The situation of target points in the N direction.

Step 2: The target points in the 8 linear directions are considered first. Taking the N direction as an example, the viewpoint (*O*) and the nearest DEM grid point (*N*_1_) have intervisibility by default (as shown in Figure 4). Thus, the points *O* and *N*_1_ determine a reference line *ON*_1_. The height (*H_i_*) of the *i*-th DEM grid point on the line defined by the point *N_i_* and viewpoint *O* is calculated by the following formula:

(1) $$H_{i}=H_{i-1}+(H_{i-1}-Z_{O})/(i-1)$$

where *H_i_* is called the critical height of the *i*-th DEM grid point and *Z* is the real height of the DEM grid point.

If $Z_{i}\geq H_{i}$, then points *O* and *N_i_* are inter-visible, and we change the reference line from *ON*_1_ to *ON_i_*; otherwise, points *O* and *N* are not inter-visible, and the line *ON*_1_ remains as the reference line.

From near to far, the visibility of all the target points in the N direction is judged in turn. Similarly, the target points in the other 7 linear directions can be judged.

**Figure 5:**
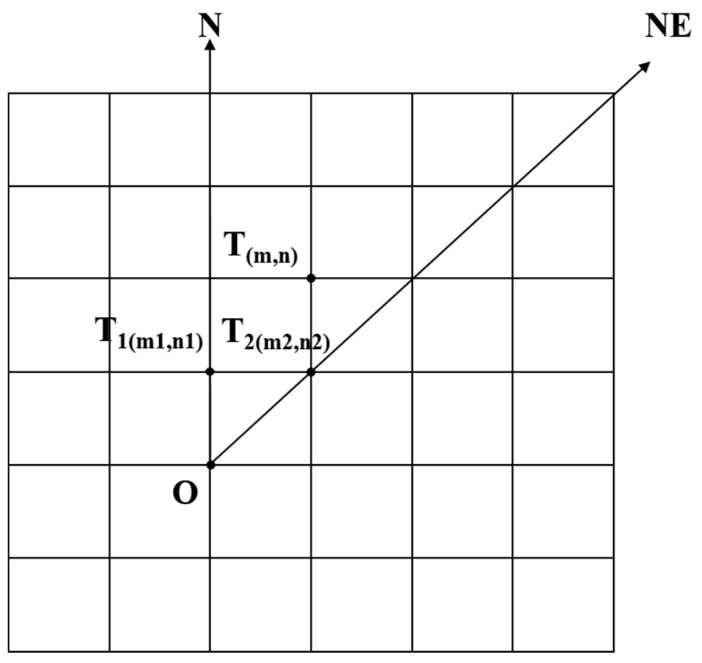
The situation of target points in N-NE sector area.

Step 3: With the target points in the linear directions being judged, the remaining target points are located in 8 sector areas. Taking the N-NE sector area shown in Figure 5 as an example, to judge the intervisibility of point *T_i_* and point *O*, we first find the two points (*T*_1_(*m*_1_, *n*_1_) and *T*_2_(*m*_2_, *n*_2_)) where *T*_1_ and *T*_2_ are in the same row or column.

The space plane equation formed by three points (*x*_1_, *y*_1_, *z*_1_), (*x*_2_, *y*_2_, *z*_2_), and (*x*_3_, *y*_3_, *z*_3_) is as follows:

(2) $$\left|\begin{array}{llll} x & y & z & 1\\ x_{1} & y_{1} & z_{1} & 1\\ x_{2} & y_{2} & z_{2} & 1\\ x_{3} & y_{3} & z_{3} & 1 \end{array}\right|=0$$

Assume $x_{ab}=x_{a}-x_{b}$, then

(3) $$z=z_{1}-[(x-x_{1})(y_{21}z_{31}-y_{31}z_{21})+(y-y_{1})(z_{21}x_{31}-z_{31}x_{21})]/(x_{21}y_{31}-x_{31}y_{21})$$

Points *O*, *T_1_*, and *T_2_* form a space reference plane *OT*_1_*T*_2_. By substituting the following values into the above formula, the critical elevation *H_i_* of point *T_i_* can be calculated.

(4) $$\left\{ \begin{array}{l} x_{1}=m_{1}\times dx;{\;y}_{1}=n_{1}\times dy;{\;z}_{1}=H_{T_{1}};\\ x_{2}=m_{2}\times dx;\;y_{2}=n_{2}\times dy;{\;z}_{2}=H_{T_{2}};\\ x_{3}=0;{\;y}_{3}=0;{\;z}_{3}=Z_{O};\\ x=m\times dx;\;y=n\times dy;\;z=H_{i}; \end{array}\right.$$

where dx and dy are resolutions in the X and Y directions, respectively.

**Figure 6:**
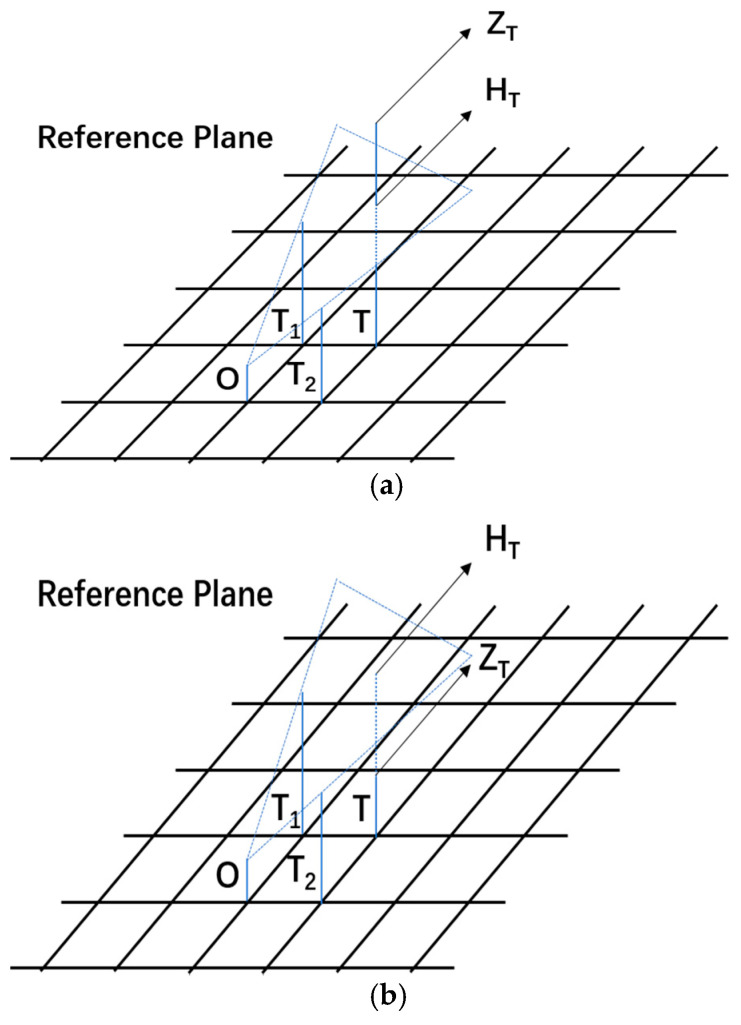
Visual judgment based on reference plane. (**a**) Points *O* and *T* are inter-visible; (**b**) points *O* and *T* are not inter-visible.

As is shown in Figure 6, if $Z_{i}\geq H_{i}$, points *O* and *T_i_* are inter-visible, and we change the reference plane from *OT*_1_*T*_2_ to *OT*_1_*N_i_*; otherwise, points *O* and *T_i_* are not inter-visible, and the reference plane *OT*_1_*T*_2_ remains as the reference plane.

From near to far, the visibility of all the target points in the N-NE sector area is judged in turn. Similarly, the target points in the other 7 sector areas can be judged.

Taking the DEM data of the area covering 100 km × 100 km as an example, it has the grid number of 3333 × 3333. In the same running environment, we use our algorithm and common algorithm to analyze the visual area. The computation time is 124.9 s (our algorithm) and 4758.8 s (common algorithm), under the Matlab running environment, showing that our algorithm is nearly 40 times faster.

### 2.2. Dilution of Precision

The accuracy of satellite positioning is related to the following two factors: (1) the measurement error of the pseudo range or carrier phase between the satellite and receiver; (2) the geometric distribution of the satellite. The geometric distribution of ground-based pseudolites is closely related to the distribution of ground-based pseudolites [20]. Precision factors are defined as follows [21]:

(5) $$\mathrm{GDOP}\;=\;\sqrt{h_{11}+h_{22}+h_{33}+h_{44}}\;=\;\sqrt{tr(H)}$$

(6) $$\mathrm{PDOP}\;=\;\sqrt{h_{11}+h_{22}+h_{33}}\;$$

(7) $$\mathrm{HDOP}\;=\;\sqrt{h_{11}+h_{22}}$$

(8) $$\mathrm{VDOP}\;=\;\sqrt{h_{33}}$$

where GDOP is called the geometric dilution of precision; PDOP is called the positional dilution of precision; HDOP is called the horizontal dilution of precision; VDOP is called the vertical dilution of precision. Matrix *H* is called the weight matrix, which is determined by satellite position and receiver position.

(9) $$H\;={\left(G^{T}G\right)}^{-1}$$

where

(10) $$G=\left[\begin{array}{cccc} -1_{x}^{\left(1\right)}(x_{k-1}) & -1_{y}^{\left(1\right)}(x_{k-1}) & -1_{z}^{\left(1\right)}(x_{k-1}) & 1\\ -1_{x}^{\left(2\right)}(x_{k-1}) & -1_{y}^{\left(2\right)}(x_{k-1}) & -1_{z}^{\left(2\right)}(x_{k-1}) & 1\\ \ldots & \ldots & \ldots & \\ -1_{x}^{\left(n\right)}(x_{k-1}) & -1_{y}^{\left(n\right)}(x_{k-1}) & -1_{z}^{\left(n\right)}(x_{k-1}) & 1 \end{array}\right]=\left[\begin{array}{cc} -{\left[1^{\left(1\right)}(x_{k-1})\right]}^{T} & 1\\ -{\left[1^{\left(2\right)}(x_{k-1})\right]}^{T} & 1\\ \ldots & \ldots \\ -{\left[1^{\left(n\right)}(x_{k-1})\right]}^{T} & 1 \end{array}\right]$$

In the formula, matrix *G* is the geometric matrix commonly used in positioning calculation, where *n* represents the number of pseudolites, and ${\left[-1_{x}^{\left(n\right)},-1_{y}^{\left(n\right)},-1_{z}^{\left(n\right)}\right]}^{T}$ represents the unit vector pointing to the i-th pseudolite from the user receiver position. The dilution of precision shows the relationship between the covariance of the positioning error and the covariance of the least squares measurement error.

## 3. Multi-Objective Particle Swarm Optimization Algorithm for Ground-Based Pseudolite Deployment

### 3.1. Mathematical Model of Multi-Target Pseudolite Deployment

The problem of multi-target pseudolite deployment is to find the optimal pseudolite distribution positions. The purpose is to obtain the highest signal coverage and the lowest average HDOP under the condition of a certain number of pseudolites. Therefore, its mathematical model can be expressed as follows:

(11) $$\{\begin{array}{l} f_{1}(X)=\mathrm{maximize}\langle \frac{S\left(X_{1},X_{2},\cdots ,X_{n}\right)}{S_{all}}\rangle \\ f_{2}(X)=\mathrm{minimize}\langle \mathrm{HDOP}\left(X_{1},X_{2},\cdots ,X_{n}\right)\rangle \end{array}$$

where $X_{1},X_{2},\cdots ,X_{n}$ refers to the location of N ground-based pseudolites; $S\left(X_{1},X_{2},\cdots ,X_{n}\right)$ refers to the visual area of the ground-based pseudolite system; $S_{all}$ refers to the whole target area; $\mathrm{HDOP}\left(X_{1},X_{2},\cdots ,X_{n}\right)$ shows the average HDOP of the visual area of the ground-based pseudolite system. Objective function $f_{1}$ refers to the problem addressed in Section 2.1, which is used to evaluate the coverage of the pseudolite system. Objective function $f_{2}$ refers to the problem addressed in Section 2.2, which is used to evaluate the positioning accuracy. Different station distribution schemes of the pseudolite system have corresponding objective function values. The best scheme is defined as the case when $f_{1}$ reaches the maximum and $f_{2}$ reaches the minimum at the same time.

### 3.2. Implementation of MOPSO Algorithm

Particle swarm optimization (PSO) is a heuristic optimization algorithm, which originates from the research on the behavior of birds. The basic idea of the PSO algorithm is to find the optimal solution through the cooperation and information sharing among individuals in the group. The MOPSO algorithm was proposed by Coello et al. (2004) [22]. The purpose is to apply the PSO algorithm that can only be used in single target to multi-target. The challenge of ground-based pseudolite system deployment is that both objective functions, i.e., maximum system coverage and minimum average HDOP, should be satisfied. Furthermore, the two objective functions $f_{1}$ and $f_{2}$ are not consistent, which may lead to the fact that the solution of this multi-objective optimization problem is not unique, but a set of optimal solutions (called Pareto optimal set [23]). The specific steps of the MOPSO process are as follows:

Step 1: Initialization of PSO parameters, such as population size, archive size, and maximum iteration number.

Step 2: According to the evaluation of the objective function, a particle swarm is randomly initialized in the decision space, including position and velocity. Suppose the number of pseudolite stations is *n*, and the coordinate of pseudolite stations *i* is $X_{i}(x_{i},y_{i},z_{i})\;(i=1,2,3,\cdots ,n)$, where $x_{i}$ and $y_{i}$ are independent variables, and $z_{i}$ can be obtained from DEM data. These *n* pseudolite stations constitute a decision vector, i.e., a particle. 

Step 3: Calculation of the fitness value of PSO. According to the Pareto domination principle, the initial archive set (also known as Pareto temporary optimal section) is obtained. The values of the objective functions $f_{1,j}$ and $f_{2,j}$ of particle *j* in the particle swarm generated after initialization are calculated. For particle *j*, if there is no objective function of other particles *k* filling the following conditions: $f_{1,k}\geq f_{1,j}$ and $f_{2,k}\leq f_{2,j}$, then particle j is put into the archive set.

Step 4: Initialization of the individual optimal particle *pbest* to itself. The global optimal particle *gbest* is randomly selected in the archive set.

Step 5: Calculation of the speed and position and their updates according to the following formula:

(12) $$\left\{ \begin{array}{l} V_{id}(t+1)=\omega V_{id}(t)+c_{1}r_{1}\left(\;pbest_{id}(t)-X_{id}(t)\right)+c_{2}r_{2}\left(\;gbest_{id}(t)-X_{id}(t)\right)\\ X_{id}(t+1)=X_{id}(t)+V_{id}(t+1) \end{array}\right.$$

where $V_{id}$ is the velocity of the particle; $X_{id}$ is the position of the particle; $c_{1}$ and $c_{2}$ are learning factors, usually set to 2, where $c_{1}$ determines its local search ability and $c_{2}$ determines its global search ability; $r_{1}$ and $r_{2}$ are random functions, and the value range is [0, 1]; ω is the inertia weight, which can be determined by the following formula:

(13) $$\omega =\omega_{\max}-\frac{\omega_{\max}-\omega_{\min}}{t_{\max}}t$$

$\omega_{\max}$ is the maximum inertia weight, usually set to 0.9; $\omega_{\min}$ is the minimum inertia weight, usually set to 0.4; *t* is the current number of iterations; $t_{\max}$ is the total number of iterations.

Step 6: According to the updated position of each particle, the fitness value of the objective function is re-calculated. The individual optimal particle *pbest* is updated on the basis of the Pareto dominance principle (PDP). Comparing the new PSO with the archive set according to the PDP, the archive set is updated, and the global optimal particle *gbest* is randomly selected and updated in the archive set.

**Figure 7:**
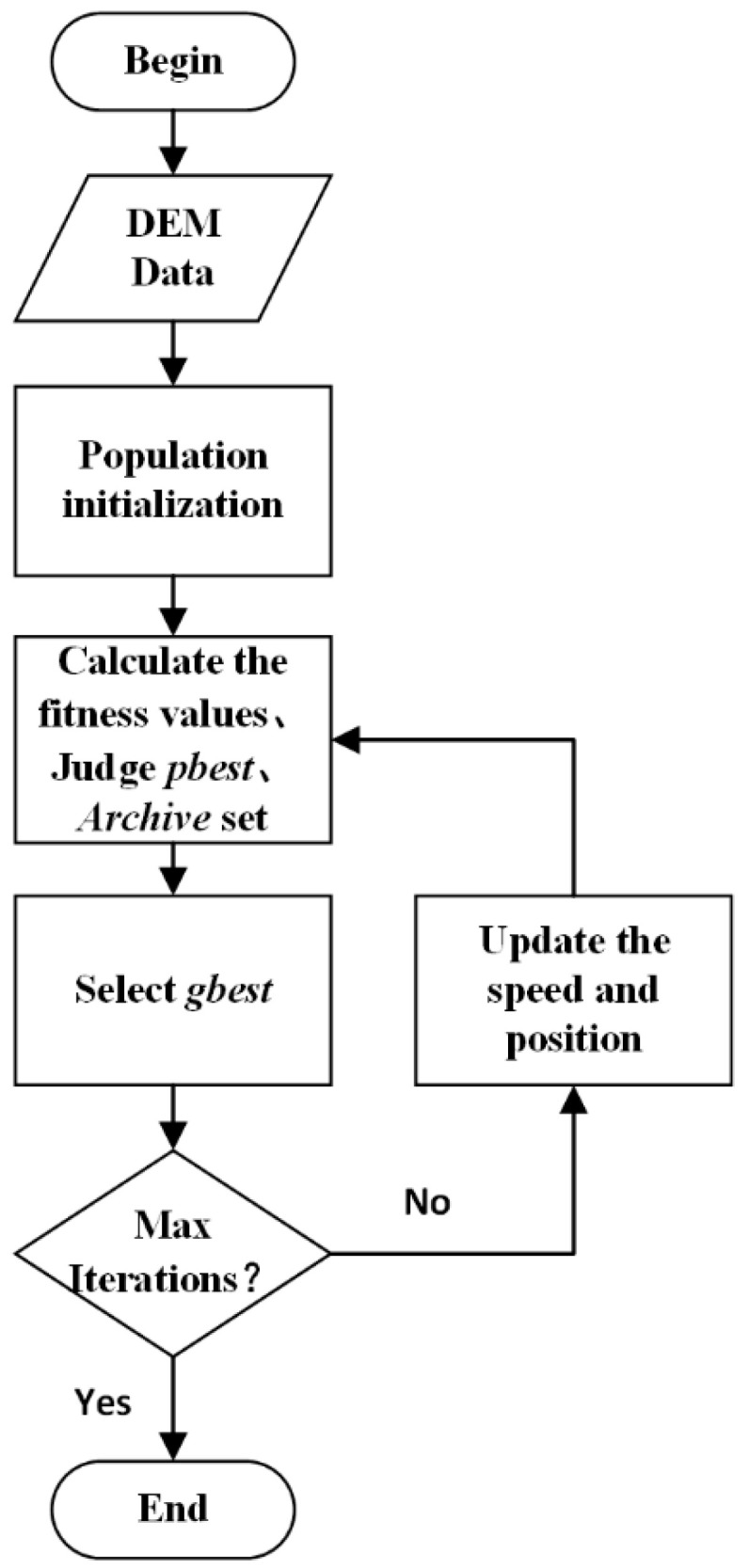
MOPSO flow chart.

Step 7: If the maximum number of iterations is reached, exit the cycle; otherwise, return to Step 5 to continue the cycle. The specific process is shown in Figure 7 below.

## 4. Simulation Experiment Analysis

### 4.1. Comparison of MOPSO and Classical PSO

The DEM Terrain Data (about 10 km × 10 km) of Jiuzhaigou area were used as an example to simulate the distribution of ground-based pseudolite stations for MOPSO. The height of the pseudolite base station was set to 10 m. The following four schemes were designed to perform comparative analysis:

Scheme 1: The stations were evenly distributed in the target area, and the number of ground-based pseudolites was set to 9, 16, 25, 36, and 49.
Scheme 2: In the target area, the classical PSO algorithm was used to optimize the signal coverage of the ground-based pseudolite system.
Scheme 3: In the target area, the classical PSO algorithm was used to optimize the average HDOP of the ground-based pseudolite system.
Scheme 4: In the target area, the MOPSO algorithm was used to optimize both the signal coverage and average HDOP of the ground-based pseudolite system.

**Figure 8:**
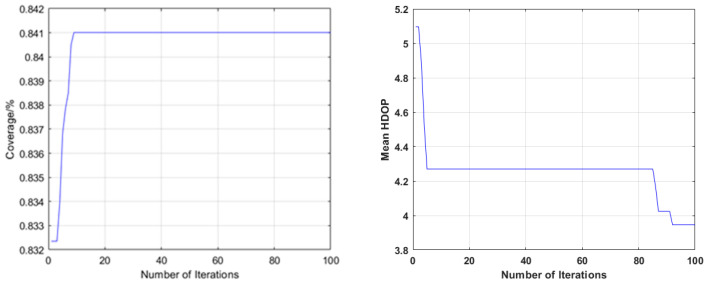
The convergence process of 16 pseudolite base stations optimized by Scheme 2 (**left**) and Scheme 3 (**right**).

Among these schemes, the parameters of Schemes 2–4 were set as follows: the population number was 50, and the maximum number of iterations was 100. Four schemes were used for simulation to obtain the relationship between coverage, mean HDOP and the number of ground-based pseudolite stations. Taking 16 ground-based pseudolite stations as an example, the convergence process of optimization using Scheme 2 and Scheme 3 is shown in Figure 8.

All the specific results of the four schemes are shown in Table 1.

As can be seen from Table 1, when Scheme 1, i.e., the average distribution of pseudolite base stations, is used, the average HDOP value is low due to the good configuration of the pseudolite distribution. However, the coverage rate is only 56.1% even if 49 pseudolite base stations are deployed. In Scheme 2, only the signal coverage of the ground-based pseudolite system is optimized, so Scheme 2 has the best system coverage in the four schemes. In Scheme 3, only the average HDOP of the ground-based pseudolite system is optimized, so Scheme 3 has the best average HDOP and the highest positioning accuracy. Scheme 4 uses multi-objective optimization; although the system coverage and average HDOP cannot be best at the same time, it achieves a good balance between the two.

**Figure 9:**
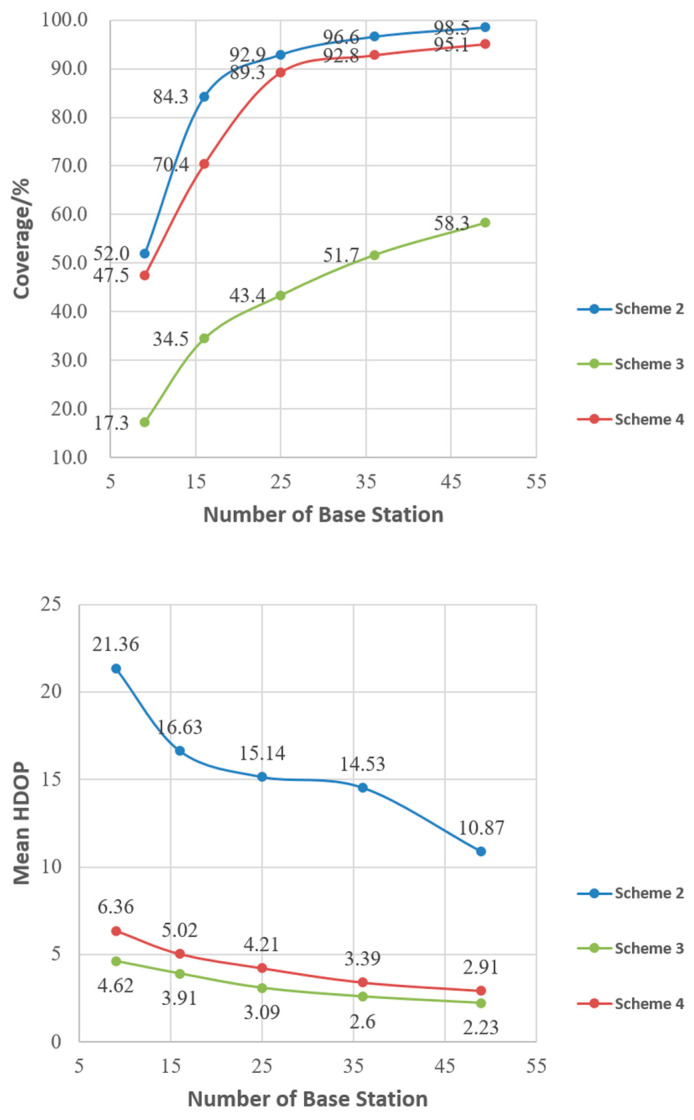
Coverage, mean HDOP, and the number of base stations under the three schemes.

As shown in Figure 9, the comparison between Scheme 2 and Scheme 4 shows that the PSO algorithm of Scheme 2 only optimizes the coverage of the pseudolite system with the single objective, while Scheme 4 needs to consider the geometry of the pseudolite system. Therefore, Scheme 4 is slightly lower than Scheme 2 in terms of system coverage with an overall difference of 7.2%. The average HDOP of Scheme 2 is 72.4% larger than that of Scheme 4, which is due to the fact that Scheme 2 does not consider the geometry of the pseudolite system. 

The comparison between Scheme 3 and Scheme 4 shows that the particle swarm optimization algorithm of Scheme 3 only optimizes the geometry of the pseudolite system with a single objective and has a smaller average HDOP. Scheme 4 needs to consider the coverage of the pseudolite system at the same time, so the average HDOP of the system is about 24.6% higher than that of Scheme 3. However, the coverage of the pseudolite system is not considered in Scheme 3, so the coverage of Scheme 4 is 49.8% higher than that of Scheme 3.

In summary, Scheme 1 adopts a uniform station distribution, which has small coverage. Both Scheme 2 and Scheme 3 adopt common single objective particle swarm optimization algorithms without considering the coverage and average HDOP of the pseudo-lite system at the same time. The proposed algorithm based on MOPSO has achieved good results in both coverage and geometry of the pseudolite system. According to the simulation results, about 25 pseudolite base stations can cover 90% of the target area.

### 4.2. Comparison of MOPSO and Convex Polyhedron Volume Optimization

To further assess the performance of the proposed MOPSO algorithm, we compared it to the well-developed convex polyhedron volume optimization (CPVO) algorithm (Nuria and Fernando, 2010) [24]. 

In the experiments, when the convex polyhedron volume optimization algorithm is used for n pseudolites, the available pseudolites distribution is first given according to the actual environment. Then, the positions of *n-1* pseudolites are fixed and the azimuth and altitude of the remaining pseudolite are adjusted.

**Figure 10:**
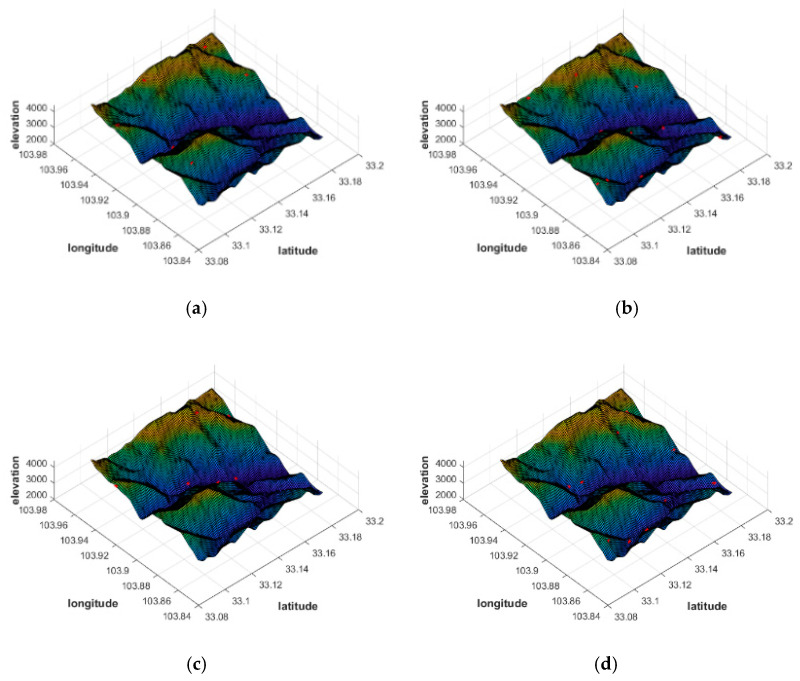
Comparison of distribution results of CPVO and MOPSO in different numbers. (**a**) CPVO 6 pseudolites. (**b**) CPVO 10 pseudolites. (**c**) MOPSO 6 pseudolites. (**d**) MOPSO 10 pseudolites.

It should be noted that for comparison, we still select the target area mentioned above, and use algorithms of CPVO and MOPSO to carry out simulation experiments. The distribution results of the two algorithms are shown in the following Figure 10 and Table 2. It should be noted that the red marked points in Figure 10 are the positions of the pseudolite base stations. Under the same number of pseudolite base stations, the two algorithms achieve different station distribution results.

From the comparison (Figure 10 and Table 2), there is no significant difference between MOPSO and CPVO algorithms in terms of mean HDOP. This indicates that the two algorithms can be both used to achieve good geometric configurations for pseudolite deployment. However, CPVO only optimizes the geometry without considering the system coverage. This results in a significant advantage of the MOPSO algorithm over the CPVO algorithm in terms of system coverage, which is about 30% higher.

## 5. Conclusions

The constellation design of pseudolites positioning is a multi-objective optimization problem with the best coverage and positioning accuracy of one certain area as the objectives. In this study, the MOPSO algorithm was used for the constellation of pseudolites. The coverage of pseudolites was determined by visible range analysis, and the average HDOP of DEM grid points was used to measure the accuracy of pseudolites positioning. The simulation results of Jiuzhaigou area in China show that:

(1). The MOPSO algorithm can optimize the geometric distribution of base stations while ensuring the system coverage.
(2). Compared with the classical PSO algorithm, the MOPSO algorithm improves the system coverage by 49.8% and the average HDOP by 72.4%.
(3). The MOPSO and CPVO algorithm both can be used to obtain good geometric configurations for pseudolite deployment. However, the MOPSO algorithm further increases by about 30% in system coverage.

The new ground-based pseudolite system deployment algorithm based on the MOPSO algorithm can not only improve the coverage of system, but also has high positioning accuracy in the coverage area, which can provide a reference for multi-target pseudolite deployment. All the above results were derived based on simulation, so the ranging capability of pseudolites, i.e., around maximum 10 km in this paper, should also be considered in real deployment.

## Figures and Tables

**Table 1:** Coverage, mean HDOP, and the number of pseudolite stations under the four schemes.

Station Number	Scheme	Coverage/%	Mean HDOP
9	1	7.7	10.07
	2	52.0	21.36
	3	17.3	4.62
	4	47.5	6.36
16	1	21.5	5.67
	2	84.1	16.63
	3	34.5	3.91
	4	70.4	5.02
25	1	32.6	4.72
	2	92.9	15.14
	3	43.4	3.09
	4	89.3	4.21
36	1	46.5	3.69
	2	96.6	14.53
	3	51.7	2.6
	4	92.8	3.39
49	1	56.1	3.19
	2	98.5	10.87
	3	58.3	2.23
	4	95.1	2.91

**Table 2:** Comparison of MOPSO and CPVO.

Algorithm	Number	Coverage/%	Mean HDOP
CPVO	6	10.1	7.83
	8	18.9	7.37
	10	22.3	7.54
MOPSO	6	40.9	6.91
	8	46.5	6.53
	10	49.3	6.15

## References

[1] Wang L., Groves P.D., Ziebart M.K. (2012). Multi-Constellation GNSS Performance Evaluation for Urban Canyons Using Large Virtual Reality City Models. J. Navig..

[2] Angrisano A., Gaglione S., Gioia C. (2013). Performance Assessment of GPS/GLONASS Single Point Positioning in an Urban Environment. Acta Geod. Geophys..

[3] Xie P., Petovello M.G. (2015). Measuring GNSS Multipath Distributions in Urban Canyon Environments. IEEE Trans. Instrum. Meas..

[4] Hsu L.T., Tokura H., Kubo N., Gu Y., Kamijo S. (2017). Multiple Faulty GNSS Measurement Exclusion Based on Consistency Check in Urban Canyons. IEEE Sens. J..

[5] Kim C., So H., Lee T., Kee C. (2014). A Pseudolite-Based Positioning System for Legacy GNSS Receivers. Sensors.

[6] Zhu X., Xu B., Li J., Nie J., Ou G. (2016). New Generation GNSS Augmentation System Based on Generalized Pseudolite. Bull. Surv. Mapp..

[7] Sheng C., Gan X., Yu B., Zhang J. (2020). Precise Point Positioning Algorithm for Pseudolite Combined with GNSS in a Constrained Observation Environment. Sensors.

[8] Kee C., Lee T., So H. (2009). Extending Operational Area of Pseudolite Using Long Integration Time and Data-less Pseudolites. Trans. Jpn. Soc. Aeronaut. Space Sci..

[9] Meng X., Roberts G.W., Dodson A.H., Cosser E., Barnes J., Rizos C. (2004). Impact of GPS Satellite and Pseudolite Geometry on Structural Deformation Monitoring: Analytical and Empirical Studies. J. Geod..

[10] Dai L., Wang J., Rizos C., Han S. (2002). Pseudo-Satellite Applications in Deformation Monitoring. GPS Solut..

[11] Meng J., Sun F., Wang A. (2007). The Research on the Plans of PL-only Positioning System. Hydrogr. Surv. Charting.

[12] Sang W., He X., Chen Y. (2013). Configuration of Pseudolite-alone Positioning System Based on DOP Geometry Structure. Bull. Surv. Mapp..

[13] Song Q., Zhang B., Li S. (2013). Study of Configuration Technology of Ground Pseudolit. Comput. Meas. Control.

[14] Fan R., Meng J., Wu M., Liu B. (2012). Distribution of the near-space pseudolite regional independent network. Sci. Surv. Mapp..

[15] Kelly R. (1990). Additional Resultson Reducing Geometricdilution of Precision Using Ridgeregression. IEEE Trans. Aerosp. Electron. Syst..

[16] Shao K., Li K., Wang J. (2017). PSO-based Pseudolite Layout Strategy. Commun. Technol..

[17] Kang G. (2006). The Study of the Pseudolite Positioning System. Ph.D. Thesis.

[18] Wang H. (2009). Research of Pseudolite Augmenting GPS Technique and Applications.

[19] Zhang B., Zhang Z., Guo L., Wang H. (2013). Key Technologies of Terrain Viewshed Analysis Based on DEM. Comput. Sci..

[20] Duerr T.E. (1992). Effect of Terrain Masking on GPS Position Dilution of Precision. Navigation.

[21] Wang W., Liu Z., Xie R. (2005). The Research On GDOP of PL-aided Beidou Positioning system. Chin. J. Space Sci..

[22] Coello C., Pulido G.T., Lechuga M.S. (2004). Handling Multiple Objectives with Particle Swarm Optimization. IEEE Trans. Evol. Comput..

[23] Zhang L., Zhou C., Ma M., Liu X. (2004). Solutions of Multi-Objective Optimization Problems Based on Particle Swarm Optimization. J. Comput. Res. Dev..

[24] Blanco-Delgado N., Nunes F.D. (2010). Satellite Selection Method for Multi-Constellation GNSS Using Convex Geometry. IEEE Trans. Veh. Technol..

